# The Role of Diet and Physical Activity in Obesity and Overweight in Children with Down Syndrome in Developed Countries

**DOI:** 10.3390/children11091056

**Published:** 2024-08-29

**Authors:** Paola Belleri, Giorgia Mazzuca, Angelo Pietrobelli, Nicola Zampieri, Giorgio Piacentini, Marco Zaffanello, Luca Pecoraro

**Affiliations:** 1 Pediatric Clinic, Department of Surgical Sciences, Dentistry, Pediatrics and Gynecology, University of Verona, 37126 Verona, Italygiorgio.piacentini@univr.it (G.P.);; 2 Pediatric Surgical Unit, Department of Surgical Sciences, University of Verona, 37126 Verona, Italy

**Keywords:** obesity, body mass index, Down’s syndrome, dyslipidemia, diet, physical activity

## Abstract

Down’s syndrome (DS), or Trisomy 21, represents the most common chromosomal abnormality in live births, characterized by an extra chromosome 21. Children affected by Down’s syndrome are more susceptible to the development of obesity and of becoming overweight compared with other children. Furthermore, they seem to present a more unfavorable lipid profile than the non-DS obese pediatric population. Diet and physical activity are closely related to the development of overweight and obesity, and they can be assessed using questionnaires such as the Mediterranean Diet Quality Index in children and adolescents (KIDMED) and the Godin–Shephard Leisure-Time Physical Activity Questionnaire. This review aims to undertake a comprehensive analysis of the intricate interplay between diet and physical activity in children affected by Down’s syndrome. Specifically, it seeks to deepen understanding regarding the question of how diet and exercise can influence and prevent the development of overweight and obesity in that special pediatric population.

## 1. Introduction

Down’s syndrome (DS) represents the most prevalent genetic disorder in live newborns [1,2]. Children with DS are affected by obesity [1]. Obesity has become one of the main concerns of global public health; it is a complex multifactorial disease resulting from the interplay and combination of several elements, including social, environmental, genetic, physiological, behavioral, lifestyle, cultural, and metabolic factors [3]. On the other hand, the risk of obesity can be decreased by interventions that combine physical activity and diet [4,5]. It is known that obesity is closely correlated to dyslipidemia [6]. The most common lipid abnormalities include hypertriglyceridemia and hypercholesterolemia [7]. Children affected by DS are more likely to become overweight or obese than the general pediatric population without DS [8]. Furthermore, DS children present a more unfavorable lipid profile than the obese non-DS pediatric population [9,10]. This comprehensive review aims to analyze the role of diet and physical activity in preventing the development of overweight, obesity and metabolic syndrome in the pediatric population affected by Down’s syndrome. Moreover, the utility of specific objective tools to assess diets and physical diets, such as the Mediterranean Diet Quality Index in children and adolescents (KIDMED) and the Godin–Shephard Leisure-Time Physical Activity Questionnaire, will be deepened.

### 1.1. Down’s Syndrome

Down’s syndrome is the most frequent hereditary cause of intellectual disability [1,11]. According to estimates, the prevalence of DS in the general population is one in 1000 to one in 2000 births [12]. The genetic abnormality of DS is the presence, either partial or total, of an extra chromosome 21 (trisomy 21) [1]. Two different genetic mechanisms can explain DS: the first involves a translocation in which an additional copy of chromosome 21 joins with an acrocentric chromosome, like 13, 14, 15, or 21 [13]. The most common translocation involves chromosomes 14 and 21 [13,14]. Therefore, an unbalanced translocation in a DS child requires a chromosomal analysis by the parents to rule out a balanced translocation. The second genetic mechanism is mosaicism, described as two or more distinct cell lines developed from a single fertilized egg [15]. In mosaic DS, not every cell has an additional copy of chromosome 21; some have an aberrant cell line with 47 chromosomes with an extra copy of chromosome 21, while some have a normal cell line with 46 chromosomes [13]. Children with Down’s syndrome may also have concomitant and complex medical issues in addition to intellectual disabilities [13]. In over half of the population with DS, hearing problems and otitis media, eye disorders, obstructive sleep apnea, and congenital heart disease may be found [9,16,17,18]. Moreover, children affected by DS are affected by thyroid abnormalities, gastrointestinal atresia, celiac disease, leukemia, iron-deficiency anemia, seizures, atlantoaxial instability, and mood and behavioral disorders at a higher prevalence than the general pediatric population [14,16,17]. Among these, it is evident that, in this population, obesity and overweight are becoming a more significant issue [9].

### 1.2. Obesity

The World Health Organization describes obesity as a chronic illness that results from an abnormal or excessive accumulation of body fat due to a positive energy balance (WHO, 1998) [19]. It can be classified depending on body mass index (BMI), percentage of body fat (PBF), waist-to-hip ratio, and bioelectrical impedance analysis (BIA) [20]. Although BMI is computed using the same formula, it is interpreted differently for children and adolescents: body weight (kg) divided by square height (m^2^) [8,21]. For children and adolescents of the same age and sex, obesity is defined as a BMI at or above the 95th percentile, whereas overweight is defined as a BMI in the 85th to less than the 95th percentile [21]. The difficulty in differentiating between fat and lean mass occasionally leads to misdiagnosis of obesity [8,22]. Analysis of per cent body fat (PBF) or bioelectrical impedance analysis (BIA) has proven more accurate in estimating fat mass, even in children with DS. However, no international pediatric standards are accepted [8,23]. In recent years, an increasing number of pediatric patients are suffering from obesity, and it has become one of the main concerns of global public health [5]. This burgeoning epidemic transcends geographical boundaries, impacting communities worldwide [5]. This condition can have several other consequences, such as cardiovascular disorders, dyslipidemia, systemic arterial hypertension (SAH), and obstructive sleep apnea syndrome (OSAS), among others [11]. Furthermore, overweight and obese children have a higher risk of maintaining this condition in adult age, perpetuating its detrimental effects throughout life [11,17]. This is precisely why early intervention is crucial to curb the trajectory of obesity and mitigate its consequences [11].

Prevention is the most effective way of dealing with chronic diseases, and children with obesity should be regarded as the target population for intervention programs: primary prevention of overweight or obesity can be combined with secondary prevention to prevent weight gain after weight loss [5]. It is essential to modify individual lifestyles and create environmental conditions that facilitate adopting and maintaining healthy behaviors over time [4,5]. Some intervention strategies that can be considered include modifications to the built environment, encouraging walking or biking to school, promoting an active lifestyle, reducing TV watching and overeating in front of the TV, and improving a prudent/healthy dietary pattern [4,5,19]. Studies have shown that the reduction of sedentary behavior, the promotion of free play and a prudent dietary pattern (based on the consumption of specific food such as whole grains, vegetables, fruits and low- and non-fat dairy) have been more effective than imposing physical activity requirements or restricting caloric intake on children [4,5,24,25,26].

## 2. Material and Methods

The electronic databases Medline PubMed Advanced Search Builder, Scopus, and Web of Science were analyzed using the following medical subject headings (MeSH) terms and text words (as well as their combinations and truncated synonyms): “Down Syndrome”, “obesity in DS children”, “overweight in DS children”, “diet”, “Mediterranean diet in DS children”, and “physical activity in DS children”. The abstracts were examined after the elimination of duplicate articles. The full text of relevant papers was analyzed. Studies providing the outcomes of case reports, case series, case-control studies, cohort studies, synthesized data, reviews, and randomized trials met the inclusion requirements. Only English-language articles were included in the research. The exclusion criteria included studies published only as abstracts, letters, conference proceedings, discussion papers, animal studies, and editorials. To find possibly relevant research, the titles were screened first and then the abstracts, before, finally, the entire article was reviewed. Two reviewers (P.B. and G.M.) independently evaluated all titles and abstracts. Quality assessments were performed by two independent reviewers (P.B. and G.M.); at the same time, the supervision undertaken by the other authors (A.P., N.Z., G.P., M.Z. and L.P.). All data were independently verified. After an initial search, an analysis based on the key questions and the inclusion and exclusion criteria narrowed down the results to 68 papers that meet the inclusion criteria.

## 3. Relationship between Obesity, Dyslipidemia and Down’s Syndrome

Abnormalities in lipid metabolism are widespread in obese patients [19]. It is estimated that between 60 and 70 per cent of those who suffer from obesity concurrently have altered blood lipid levels [19]. High blood levels of triglycerides represent the most common dyslipidemias, increased total cholesterol and LDL cholesterol blood levels and a reduction in high-density lipoprotein (HDL) cholesterol [19,27]. Moreover, gamma-glutamyl transferase (GGT) should be routinely evaluated in this context as it significantly correlates with increased risk of obesity, systemic arterial hypertension, and dyslipidemia [9]. Children affected by DS are more likely to become overweight or obese and to present an unfavorable lipid profile than the general pediatric population without DS. Elevated total cholesterol, LDL cholesterol, triglycerides, and decreased HDL cholesterol levels are often observed [10,13]. This highlights the intricate interaction between metabolic dysregulation and typical abnormalities in patients with DS [28,29].

### 3.1. Obesity and Down’s Syndrome

The diagnosis of obesity in patients with DS is formulated using anthropometric measurements, in particular, BMI analysis or PBF, and related percentiles are calculated using specific and special growth charts [21]. Specialized growth charts are used to precisely assess the growth and nutritional status of children with DS [30,31,32]. DS growth charts have been issued in several countries, even if the American Academy of Pediatrics recommends using the WHO and CDC standard growth charts for patients with DS [30,33].

Indeed, stratifying patients according to PBF leads to a significantly lower prevalence of obesity and overweight compared with the BMI z-score curves (31% vs. 57.9%) [34]. The DONUT study examined this special population further and compared these two variables in a small sample of patients with DS (*n* = 33) from a single center in northern Italy [8]. The study demonstrated a higher prevalence of overweight and obesity in children with DS compared with peers without DS and a lower prevalence when stratifying patients according to PBF compared with the BMI z-score curves (42% vs. 54.6%) [8]. Therefore, it suggests that although BMI continues to be the primary method for identifying obesity in children with Down’s syndrome., it may slightly overestimate the level of obesity compared with PBF in DS children [8,9]. Several factors are crucial for the development of overweight and obesity in children affected by DS [10,11,25]. Despite the syndrome’s intrinsic physical and physiological characteristics, muscular hypotonia and ligament laxity emerges, thus leading to excessive flexibility and structural abnormalities, congenital heart diseases, reduced respiratory capacity (reduced peak oxygen consumption), hypothyroidism, decreased basal metabolic rate, and macroglossia [2]. In addition, developmental delays and cognitive impairment may limit participation in physical activities and influence adherence to recommended nutritional plans, leading to inadequate dietary habits. This condition can contribute to weight gain [25,35]. In this context, employing a multifaceted approach involving preventive measures, lifestyle modifications, and targeted interventions can lead to significant change and improve the substantial impact of obesity on the pediatric population, particularly for children affected by DS, and on global healthcare systems [11,27]. Several factors have been identified as possible causes of significant weight gain in children with DS, such as untreated hypothyroidism, elevated leptin levels, decreased energy expenditure at rest, limited physical activity and unhealthy diet [36]. To prevent this condition, the DS population must undergo systematic nutritional evaluations during childhood and adolescence [36]. Generally, DS children present adequate compliance to a Mediterranean diet, which could be considered a healthy dietary pattern, depending on the geographical area of origin—Spain has the highest adherence rate [9,36]. On the other hand, DS children are less sedentary. Early nutritional education for families and caregivers should be a component of these prevention strategies [37].

### 3.2. Dyslipidemia in Down’s Syndrome

Compared with the pediatric population without DS, children with DS may exhibit a more unfavorable lipid profile [2,8,9]. Several mechanisms can explain this association: although lifestyle habits may influence lipid profiles during later stages of life, their role as a consolidated causal factor in DS remains unverified, strengthening the hypothesis of an underlying genetic mechanism driving lipid profile alterations in individuals with DS [14,38]. Previous research has identified a susceptibility locus on chromosome 21 associated with elevated Apo B levels, a primary constituent of very low-density lipoproteins (VLDL) and LDL cholesterol [38]. More recently, it was found that chromosome 21, specifically in the 21q11 region, contains a gene locus responsible for encoding a VLDL receptor, perhaps serving a crucial function in controlling lipid metabolism [39]. Additionally, increased cholesterol levels have been observed in fetuses with trisomy 21 during intrauterine development, suggesting that lipid metabolism abnormalities could influence lipid levels before other factors [39,40]. Despite this, the relationship between dyslipidemia and DS is still conflicting [11]. It has been demonstrated that the most frequently observed alterations are a reduced HDL cholesterol level (12.5%) and alterations in total cholesterol (9%) [2]. It has also shown an interesting inverse correlation between HDL values and PBF: for every point increase in PBF, there is a decrease in HDL cholesterol value of 0.6 mg/dL [2,8]. No significant changes in lipid profile have been shown [2]. Obesity and dyslipidemia are significantly related to non-alcoholic fatty liver disease (NAFLD). Currently, NAFLD is considered the most frequent chronic liver disease worldwide. According to epidemiological research, nonalcoholic fatty liver disease (NAFLD), a disorder marked by excessive fat buildup in the liver, is becoming more common in children as well as more obese children [41]. In children with DS, an increased prevalence of non-alcoholic fatty liver disease (NAFLD) has been observed. According to a recent Italian study, the incidence of nonalcoholic fatty liver disease increased by 45% in children with DS who were of normal weight compared with their peers who had similar features. In overweight or obese participants with DS, the prevalence increased to 82% [8,41]. One explanation for this could be the altered production of adipocytokines, leading to higher blood levels of leptin, adiponectin, IL-6, and TNF-α [41]. Furthermore, the study corroborates existing literature findings concerning alterations in liver function profiles, particularly emphasizing the discernible correlation between elevated levels of alanine aminotransferase (ALT) and GGT and the body mass index (BMI) z-score or per cent body fat (PBF) [8]. These findings highlight how crucial it is to exercise caution and adopt proactive health management techniques as early screening protocols to detect hepatic steatosis and reduce the risk of liver issues in children with Down’s syndrome who are overweight or obese [8].

## 4. Intervention Strategies for Childhood Obesity in Down’s Syndrome

Obesity is a multifactorial disorder due to its modifiable and non-modifiable risk factors [5,19]. The research has repeatedly demonstrated that diet and physical activity are strongly correlated with the development of overweight and obesity [5]. They are the main modifiable risk factors influencing that condition [5]. Studies have shown beneficial benefits in the short and long terms, depending on a combination of dietary changes, behavioral therapy, and physical activity for childhood obesity [5,6]. The cornerstone of treatment is a diet tailored to the patient’s age. Appropriate calorie intake, optimal nutrition for maintaining health and normal growth, and assistance in developing healthy eating habits represent the principal nutritional goals. Nevertheless, regular physical activity is essential to prevent abnormal weight gain and sustain that prevention [42].

### 4.1. Mediterranean Diet as an Example of a Healthy and Balanced Diet

Children with DS have selective nutrition due to intrinsic characteristics of the syndrome and family dietary habits, which may also vary between geographical origins [10,43]. Clinical characteristics, such as hypotonia, limited oral space, sluggish swallowing reflex, and impaired food perception based on consistency, taste, warmth, and fragrance, contribute to the development of improper eating habits in children with Down’s syndrome [43]. Additionally, hypotonia impairs the strength and movement of the mouth muscles, which can lead to problems with swallowing, chewing, lip closure, tongue protrusion, and reflux of the stomach [9,17,43]. Pasta, bread with jam or bologna or butter, milk, packaged juice, mixed fruit, cookies, desserts, soft drinks, flour, and tubers represent the most widely consumed food [2,25,44]. These food choices lead to an excessive consumption of the daily recommended quantities of protein, carbohydrates, and lipids, thus increasing their energy and macronutrient intake. In contrast, they should consume more unsaturated omega-3 and omega-6 fatty acids and vitamins and minerals such as A, B9, B12, E, zinc, magnesium, iodine, and selenium [2]. Finally, a systemic overview analyzing micronutrient status in children and adolescents with DS has demonstrated that comparatively few consistent studies have been conducted in this field. More well-planned clinical trials are needed to investigate the micronutrient status and impact of dietary supplements in children with Down’s syndrome [45]. For this reason, it is crucial to establish a healthy and feasible diet for children with DS [2]. The Mediterranean diet is often recommended as a good health principle, lifestyle, and cultural pattern, and it is the world’s most documented and well-known dietary pattern [2,46]. This diet is characterized by the consumption of an extensive range of foods, including olive oil, fish, dairy products, legumes, grains, nuts, fruits, and vegetables [47]. A pyramid is used to illustrate recommended portion amounts and consumption frequencies. At the base of the pyramid are items that should be eaten at each meal in addition to the Mediterranean diet’s social, cultural, and environmental aspects [48]. These foods include two or three portions of bread, pasta, couscous, rice, oats, other daily grains, and fruits and vegetables [46,47]. Items that should be eaten every day are grouped in the middle of the pyramid: two servings of milk and yoghurt, one dish of breakfast items such as cereal and cookies, two to three portions of extra virgin olive oil as the primary source of fat, and lastly, herbs and spices for seasoning [47,48]. Higher up the food pyramid are those that should be eaten once a week, such as animal products, which should be replaced with legumes and consumed with cereals [48]. Potatoes ought to be consumed once a week as well. Sugary foods like these belong at the top of the pyramid and should only be consumed once a week [48]. Data have firmly established that nutrition type is a critical factor in promoting a healthy lifestyle, reducing adiposity indices and preventing many chronic disorders such as diabetes, cardiovascular diseases, obesity and overall mortality [46,47,49,50].

### 4.2. Physical Activity

Physical activity represents an important modifiable factor that can influence the development of overweight and obesity, enhancing many health indices, such as overall quality of life, brain health, cognition, memory, sleep, and anxiety [51,52]. WHO guidelines include worldwide recommendations on the amount of physical activity for various age and population groups [52]. Guidelines advise 60 min of moderate-to-intense physical activity thrice weekly [52]. Despite the well-known beneficial effects of physical activity, children and adolescents with DS are more susceptible to musculoskeletal and cerebral impairments, which might affect their motor function [53]. Therefore, the risk of physical inactivity and obesity is very high [54]. Children with DS are less sedentary than their peers and often fail to achieve this target [52,55]. Physical exercise in clinical settings could promote, enhance, or restore physical health and musculoskeletal function. Exercise may also have a favorable impact on any body system, particularly aerobic exercise with a frequency of 5 days, a duration of 6–8 min and an intensity of between 0.2 and 0.5 m/s [56]. Moreover, another study conducted on 77 children with DS compared with 57 children without DS underlines that those with DS participated in more minutes of light physical activity (LPA) (*p* < 0.0001), fewer minutes of sedentary activity (*p* = 0.003), and a tendency toward fewer minutes of moderate-to-vigorous PA (MVPA) (*p* = 0.08) [55,57]. Therefore, encouraging children with DS to practice LPA may provide a workable plan for reaching a healthy weight [57]. Finally, promoting participation in physical activities, even in inclusive environments, such as schools or after-school centers, promotes social interaction for children with DS and develops relationships with their peers. This can promote an inclusive and positive culture around physical activity [9,51,58,59].

### 4.3. KIDMED and GODIN Questionnaires as Healthcare Interventions

The literature has long established a strong correlation between obesity’s modifiable risk factors, diet, and physical activity [51]. There are different methods by which to assess food consumption in order to provide a healthy lifestyle, including the Food Frequency Questionnaire (FFQ) or Mediterranean Diet Quality Index in children and adolescents (KIDMED). FFQ is a retrospective method for assessing food consumption [2,26,37]. A modified FFQ has been utilized to evaluate the frequency of consumption across 62 product groupings in children with DS; however, it was country-specific, and the portion size needed to be considered [26]. Due to this, in our review, the KIDMED questionnaire has been identified as a possibly more accurate method by which to assess food [2,26,37]. The GODIN questionnaire has been chosen as the most appropriate method to evaluate physical activity [9].

#### 4.3.1. KIDMED Questionnaire

**Figure 1:**
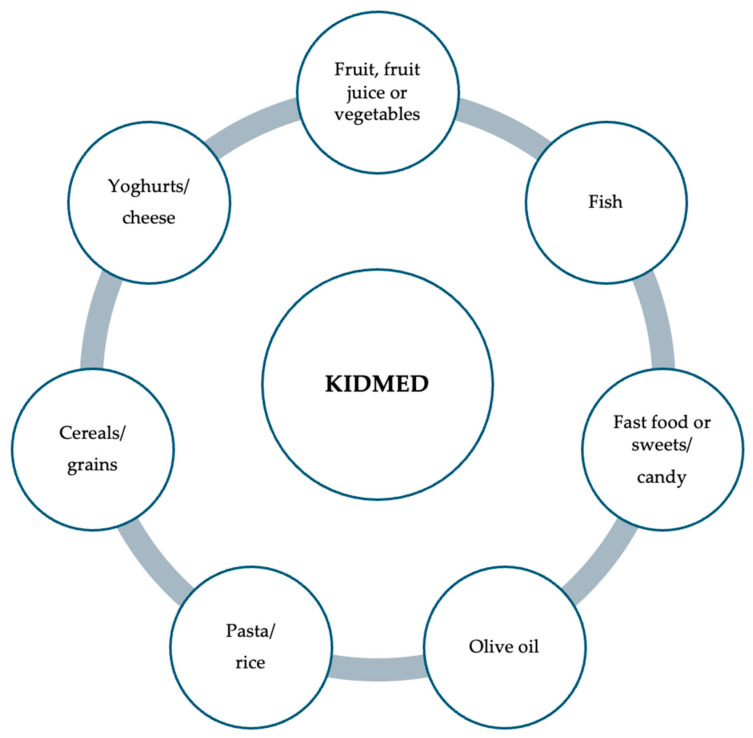
Items examined as part of the children’s and adolescents’ “Mediterranean Diet Quality Index” questionnaire.

Adherence to the Mediterranean diet can be assessed through pediatric questionnaires, with the Mediterranean Diet Quality Index in children and adolescents (KIDMED) being the most prominent among them [60]. The KIDMED index was developed using factors that support and oppose Mediterranean eating patterns. Based on a 16-question test, the index ranged from 0 to 12 and may be self-administered or completed via interview [60]. Questions that reveal negative information about the Mediterranean diet receive a score of 21, while those with a positive aspect receive a rating of +1 [60]. The following three levels were identified based on the sums of the test results, depending on patient adherence: 8, good adherence, the ideal Mediterranean diet; 4 to 7, average adherence, improvement is needed to adjust consumption to match Mediterranean patterns; <3, poor adherence, deficient quality [60,61] (Figure 1).

Generally, moderate adherence to the Mediterranean diet was recorded in children with DS (65%), followed by high and low adherence [9]. Through the examination of KIDMED questionnaire outcomes, it becomes evident that the degree of adherence to this dietary regimen predominantly hinges upon entrenched family customs rather than the individual dietary inclinations of the child [9,60,62]. This underlines the significant influence of familial nutritional practices and the importance of familial dietary patterns in shaping children’s eating habits and nutritional choices [9]. The DONUT study, analyzing patients with a 2-year follow-up, demonstrates no substantial changes in diet adherence scores between the beginning and end of the study [9]. It has been shown that compliance to a Mediterranean diet depends on the geographical area of origin, both in patients with DS and in peers without DS; adequate adherence to the Mediterranean diet was observed for the Italian pediatric population, regardless of whether the child has DS [63]. Moreover, the adherence for the Italian pediatric population is higher compared with some European states (Crete, Cyprus, Greece) but lower if compared with the Spanish pediatric population, which remains the top-ranking in adherence [64,65,66]. Furthermore, variations within the Italian pediatric population have been highlighted based on geographical location, with discernible differences noted depending on the region of origin (higher in the northern Italy pediatric population than their counterparts in southern Italy) [67]. This regional disparity in dietary habits suggests that cultural and environmental factors influence dietary preferences and patterns. Because there are no significant differences between children with and without DS, it is recommended that guidelines be created that are suitable for a general pediatric population to prevent the development of obesity [9,63].

#### 4.3.2. Godin–Shephard Leisure-Time Physical Activity Questionnaire (GODIN)

**Figure 2:**
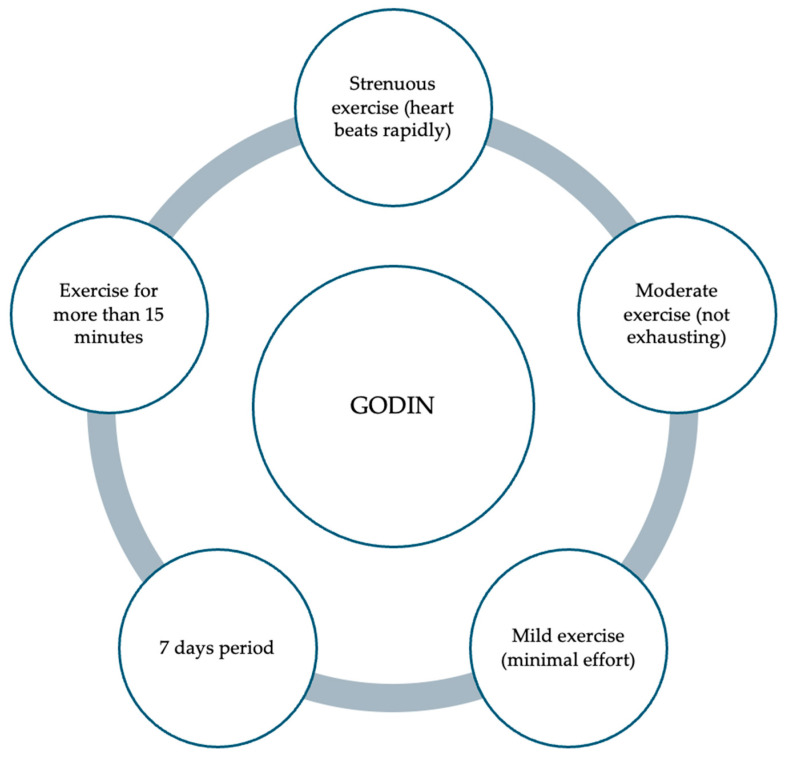
Items examined with the “Godin–Shephard Leisure-Time Physical Activity Questionnaire”.

The quantification of physical activity levels can be effectively assessed through various methodologies, including validated questionnaires such as the Godin–Shephard Leisure-Time Physical Activity Questionnaire (GODIN) [68]. The GODIN questionnaire comprises a structured assessment tool encompassing four distinct items, with the questionnaire’s first three questions intended to gather information about the frequency with which individuals engage in leisure-time physical activities of varied intensity levels. In particular, participants are asked to specify how often they engage in light, moderate, and intense physical activity sessions that last at least 15 min in an average week (Figure 2) [68]. By employing such instruments, researchers and healthcare professionals can gain comprehensive insights into an individual’s activity patterns, thereby facilitating tailored interventions to promote optimal health and mitigate the risk of obesity development [68]. The GODIN questionnaire was used in the DONUT study to quantify physical activity, with moderate activity being the most frequently registered, followed by low and high activity [9]. No studies have analyzed the level of physical activity in children with DS or general pediatrics. This gap in research can be attributed to the limited utilization of appropriate pediatric-specific questionnaires, such as the Godin questionnaire, designed to capture physical activity levels in children accurately [9,68].

For this reason, it is not easy to compare the Italian population with populations from other states [2,8,9]. Furthermore, in another study, regular outpatient follow-up was undertaken in patients with DS for about 2 years (2020–2023) to assess whether these factors were effectively modifiable through counselling and patient management [2]. The study sample consisted of 44 children from a single center, all with DS. The study shows that the average KIDMED score remained stable at 6 out of 12 at the beginning and end of the study [2]. However, the average GODIN score underwent a significant change, increasing from an initial average score of 17 to 22 points at the end of the study [9]. This led to a change in the lifestyle of children with DS, who were committed to a less sedentary and more active life with moderate-to-vigorous physical activity [9]. This average score difference demonstrates how counselling results in actual improvement [7]. Moreover, the DONUT study highlights the relationship between increased physical activity and variations in biochemical parameters, especially HDL cholesterol [8]. Furthermore, it shows a lower prevalence of obesity at the end of the follow-up period, as indicated by a decrease in the BMI z-score [8]. These data show that the GODIN questionnaire is useful for predicting anthropometric variables and laboratory alterations [2].

## 5. New Strategies: A Tailored Ambulatory Pathway

Routine visits are essential for preventing overweight and obesity, especially in children with DS [2]. Establishing a dedicated outpatient pathway tailored to the needs of parents of children with DS holds promise in delivering essential counselling and support [2]. This pathway would prioritize attention to anthropometric measurements and the conducting of regular laboratory tests, ensuring a holistic care approach [13]. Previous research has highlighted that parents with inadequate nutritional education may improperly manage their diet and nutrition, which can influence the food choices of children affected by DS [28,29]. Moreover, it is imperative to provide counselling that emphasizes the critical role of physical activity, recognizing that familial sedentary behaviors can impact the child [54,62]. Therefore, educating parents on the importance of an active lifestyle becomes fundamental [2,54]. The KIDMED and GODIN questionnaires, besides being readily available tools, can be valid and useful in the clinical management of patients with DS, making it easier to identify individuals at risk [2,9].

## 6. Conclusions

The management and prevention of the development of obesity and overweight is crucial, especially for such a vulnerable class of patients, such as those with Down’s syndrome. Educating and sensitizing the family of these patients is fundamental to promoting a healthy lifestyle that includes a proper diet, nutrition, and adequate physical activity. This could allow for an overall improvement in the metabolic health of the Down’s syndrome patient. Early intervention strategies, including stimulation therapy and behavioral interventions, are the cornerstone for managing children affected by DS, especially for improving long-term outcomes.

## Data Availability

Not applicable.
